# Evaluation of Antimicrobial Activity and Cytotoxicity of an Epoxy Resin-Based Endodontic Sealer Containing Nanoparticles Amorphous Calcium Phosphate

**DOI:** 10.1155/2023/8717655

**Published:** 2023-12-05

**Authors:** Bilal H. Ibrahim, Hussain Al-Huwaizi

**Affiliations:** Aesthetic and Restorative Dentistry, College of Dentistry, University of Baghdad, Baghdad, Iraq

## Abstract

**Background:**

The main cause of posttreatment disease in endodontics is the remaining of microorganisms within the root canal walls after endodontic therapy. Therefore, it is essential to use root canal sealers with potent antibacterial properties. These sealers play a vital role in eradicating any remaining microorganisms and preventing recontamination, especially in situations where there might be microleakage.

**Aim:**

The objective of this research was to examine the antimicrobial properties of epoxy root canal sealers containing nanoparticle amorphous calcium phosphate (NACP) against planktonic *Enterococcus faecalis* in a controlled laboratory environment. Furthermore, the study aimed to assess the potential cytotoxic effects of these sealers.

**Method:**

In order to determine the antimicrobial activity, the epoxy resin sealer (AH Plus, Dentsply, Germany) was supplemented with NACP from Sigma–Aldrich, at a concentration of 3wt.%, as per the previous flowability tests. The agar well diffusion assay method was employed to evaluate the antibacterial efficacy. For this, cultured plates (*n* = 8) were prepared, with each plate containing three wells: one with AH Plus, one with AH Plus + NACP, and one with NACP alone. Subsequently, the plates were sited at 37°C in an incubator and allowed to incubate overnight. The width of the inhibition zones was then analyzed and recorded by the SPSS statistical software package (Version 20.0 for Windows, SPSS, Chicago, IL, USA). The cytotoxicity of the NACP incorporated AH Plus and AH Plus sealers were tested indirectly by MTT assay and directly by the multiparametric high content screening toxicology assay using fibroblast-like cells as cell lines.

**Results:**

AH Plus + NACP showed a higher antimicrobial activity than AH Plus with significant difference (*P* < 0.0001). Both materials showed nonsignificant difference compared to negative control, which indicated lower cytotoxicities. For AH Plus, *P*=0.3599, 0.5959,  and 0.4071, with time intervals 24, 72, and 168 hr, respectively. For AH Plus + NACP, *P*=0.4386, 0.6182,  and 0.2080, with 24, 72, and 168 hr, respectively.

**Conclusions:**

NACP incorporation with epoxy resin sealer AH PLUS had a higher antimicrobial activity with lower cytotoxic effect indicating their potential therapeutic values.

## 1. Introduction

To achieve a successful endodontic treatment outcome, it is crucial to effectively do cleaning and shaping of the root canal space and then fill it three-dimensionally obturation with a hermetic seal [1, 2]. Endodontic failure or posttreatment disease typically arises when bacteria and their byproducts remain within the root canal system. This presence of bacteria can result from inadequate cleaning and shaping of the system or from potential leakage from the oral cavity. Incomplete cleaning and shaping of the canals or oral cavity leakage can lead to the presence of bacteria [1].

While no filling material is perfect, for many years, a combination of gutta-percha and sealer has been widely regarded as the most effective root canal filling material [3].

During the early 2000s, the emergence of adhesive dentistry to endodontics led to the development of an innovative type endodontic sealers that is polymer-based that could effectively bond to the root canal dentin [3, 4]. These resin-based sealers create hybrid layers, thanks to their properties of hydrophilicity, allowing them to infiltrate deeply into the dentinal tubules [3]. The adhesion of these sealers depend on the interaction between the sealer and the root dentin [5].

Resin-based sealers primarily fall into two categories: epoxy-based and methacrylate-based [3]. Among these options, epoxy resin-based sealers have garnered extensive approval in the field of endodontics due to their favorable characteristics. They offer extended working time, making them convenient to use, are easily mixed for efficient applications, and exhibit excellent sealing capabilities, ensuring effective root canal obturation [6, 7].

Resin-based sealers were introduced to the market to address the shortcomings of traditional sealers. One notable example is AH Plus, a root canal therapy epoxy resin-based paste–paste sealer system that has gained widespread acceptance. This system comprises an epoxy paste and an amine paste containing three distinct types of amines. AH Plus has been shown to exhibit excellent resistance to solubility and maintain stable dimensions when exposed to various solutions [8].

Additionally, it possesses the ability to adhere effectively to dentin, attributed to its properties of creep, and extended time of setting [8–10].

Concerning antimicrobial attributes, sealers that are epoxy resin-based demonstrated antibacterial effects, primarily prior to their set, owing to the discharge of certain ingredients, such as formaldehyde [11, 12].

Numerous attempts undertaken to extend the antimicrobial capabilities of epoxy-resin sealers by incorporating antibacterial agents like silver, chlorhexidine, quaternary ammonium compounds, calcium hydroxide, and various others. These additions have demonstrated enhanced antimicrobial efficacy while causing minimal harm to the physical, chemical, and biological characteristics [13].

Methacrylate-resin-based endodontic sealers were presented to provide the concept of a “mono-block” by bonding the core filling material to the canal wall and forming one entity [14]. They were innovated to provide a better seal and mechanically reinforce affected roots, which have been suggested to reduce bacterial entry paths and strengthen the root assembly [8, 14, 15].

Methacrylate-based sealers are meant to infiltrate the moderately demineralized collagen matrix and create micromechanical retention to root dentin [12, 14]. While mono-block idea is appealing, the inherent challenge lies in the inability to alleviate polymerization shrinkage stresses, which stem from the unfavorable root canal cavity structure. Consequently, dislodging of resin tags from the dentinal tubules that caused by these stresses. This, in turn, jeopardizes the integrity of the dentin-sealer bond and gives rise to microgaps that serve as potential sources of microleakage [12, 14].

The use of irrigation solutions like sodium hypochlorite (NaOCl) and ethylene diaminetetraacetic acid (EDTA) during root canal preparation can have detrimental effects on root dentin structure. Earlier studies have verified that these solutions can alter the levels of essential calcium (Ca) and phosphate (P) ions in dentin, ending with mechanical impairment, decreased dentin hardness, and an elevated risk of root fractures [16, 17].

Nanoparticles incorporation of amorphous calcium phosphate (NACP) into resins has shown promising results by increasing the release of calcium and phosphate ions [18, 19]. These ions play a crucial role in restoring lost minerals and have the potential to enhance the mechanical properties of root dentin. Furthermore, transmission electron microscopy (TEM) images from previous studies have revealed the unique structure and distribution of NACP within dentinal tubules [20].

NACP, thanks to their increased surface area and superior release of calcium and phosphate ions compared to microsized counterparts, effectively facilitated the in vitro remineralization of both enamel and dentin lesions. This was achieved without compromising the material's essential mechanical and bonding properties [18, 21].

Prior TEM images have depicted NACP's capacity to seamlessly integrate with dental resins, infiltrating dentinal tubules, all the while emitting substantial quantities of calcium and phosphate ions [22].

The main goal of root canal treatment is to eradicate microorganisms from the root canals and establish an effective seal at both the apex and coronal regions. By eliminating the presence of microbes within the canal, the goal is to minimize or eliminate the risk of reinfection in the future [23–25]. This tight seal and microbial elimination are essential for the long-standing success of the root canal therapy.

The successful eradication of microorganisms in endodontic therapy requires the implementation of suitable chemomechanical instrumentation, intracanal medication, and the utilization of root canal filling materials. These elements work together to create a protective barrier, preventing microbial penetration from the oral cavity or periapical tissues. By employing these strategies, the aim is to ensure the effective removal of microorganisms and establish a favorable environment for the healing and long-term success of the treated tooth [26, 27].

Histological evaluations of failed endodontic treatments have identified the persistence of microorganisms inside the root canal system as a primary cause of treatment failure [23, 28]. The intricate structure of the root canal system and the ability of biofilms to resist disinfecting agents are major factors that contribute to this challenge [28, 29]. Despite treatment efforts, microorganisms can remain within the intricate structures of the root canal system, leading to persistent infection and potential treatment failure. Addressing these challenges is crucial to improve the success rates of endodontic procedures.

Extensive research has focused on the role of *Enterococcus faecalis* (*E. faecalis*) in posttreatment disease. Through the application of polymerase chain reaction detection techniques, studies have consistently identified *E. faecalis* in a significant percentage of failed endodontic cases, ranging from 67% to 77% [30, 31]. This type of Gram-positive bacteria has the capability to penetrate dentinal tubules, attach itself to collagen, and endure extended periods of nutrient scarcity, which can contribute to its resistance against chemomechanical instrumentation and intracanal medication [30, 32, 33]. The presence and resilience of *E. faecalis* highlight the importance of effectively targeting and eliminating this microorganism to enhance the success rates of endodontic treatment.

One highly desirable characteristic in root canal sealers is their strong antibacterial effectiveness, as it play a crucial role in eradicating residual microorganisms and preventing recontamination, particularly in cases of microleakage. Previous research has indicated that the majority of endodontic sealers exhibit varying degrees of antibacterial properties [12, 34]. Nevertheless, there is a growing interest in the root canal sealer improvement that possesses strong and durable antibacterial properties. Such a sealer holds great potential in enhancing the effectiveness of endodontic therapy and minimizing the likelihood of tooth loss in subsequent periods.

Until now, no research has investigated the integration of NACP to an endodontic sealer to achieve robust and persistent antibacterial properties, as well as the ability to calcium and phosphate ions discharge. Therefore, the primary objectives of this investigation were twofold: (1) to progress an endodontic sealer that incorporates NACP, enabling it to possess antibacterial and remineralization capabilities while maintaining other essential sealer properties; and (2) the study aimed to assess the antibacterial efficacy of the sealer against *E. faecalis* and evaluate the cytotoxicity of incorporating NACP into the AH Plus root canal sealer.

## 2. Materials and Methods

### 2.1. Preparation of Samples

Commercial endodontic sealer (AH Plus, Dentsply) was used in this study, and nanoparticle amorphous calcium phosphate (NACP) was previously made by Sigma–Aldrich, nanopowder < 150 nm particle size (BET), the details of these materials are shown in Table 1. The powder was incorporated to a freshly mixed sealer with a ratio 3wt.% according to a previous pilot study (flawability test) that not adversely affect the physical properties of the sealer.

### 2.2. Antibacterial Activity

To evaluate the antibacterial activity, the prepared samples (AH Plus, AH Plus + NACP, NACP) were subjected to the agar well diffusion assay against *E. faecalis* bacterial strains [35, 36]. Sterile Petri dishes were filled with 20 mL of Muller–Hinton (MH) agar in aseptic conditions. The bacterial strains were obtained from their stock cultures, specifically the *E. faecalis* strain CE_12_3 with a 16S ribosomal RNA gene sequence ID of MN629256.1, using a sterile wire loop [37]. Following bacterial culturing, three wells with a diameter of 6 mm were created on each agar plate (eight plates) using a sterile tip. The different samples (AH Plus, AH Plus + NACP, NACP) were then placed into these wells. NACP was added to a freshly mixed AH Plus sealer at a concentration of 3wt.% and mixed consistently using a disposable plastic spatula attached to a cordless drill [38]. NACP was diluted with distilled water (3wt.%) and injected into the wells. After incubating, the cultured plates containing the samples (AH Plus, AH Plus + NACP, NACP) and the test organisms overnight at 37°C, the average size of the inhibition zones was measured with millimeters and documented [39, 40].

#### 2.2.1. Statistical Analysis

Statistical analyses were conducted with the SPSS statistical software (Version 20.0 for Windows, SPSS, Chicago, IL, USA). Quantitative data are presented as mean, standard deviation, minimum, and maximum values. The differences between the three studied zones of inhibition were assessed using an analysis of variance test (ANOVA) and the least significant difference (LSD) method. A significance level of <0.05 was employed.

### 2.3. Cytotoxicity Studies Top of Form

Cytotoxic assays serve as primary screening tests for assessing the biocompatibility of the tested materials. The cytotoxicity of the 3% NACP incorporated AH Plus sealer in comparison to the control sealer (AH Plus) were tested indirectly by MTT assay (indirect contact test by exposing the cells to the adhesive extracts eluted from them) and directly by the multiparametric high content screening toxicology assay (through direct contact of cells with the NACP) using the fibroblast-like cells as cell lines. The experiments for both assays were carried out at the University of Malaya Centre of Natural Product Research & Drug Discovery in Malaysia.

The cell line employed in this study consists of Primary Dermal Fibroblast Normal; Human, Neonatal (HDFn), PCS-201-010™. HDFn, derived from neonatal foreskin, exhibits fibroblast-like characteristics and finds utility in various research areas, such as pathogen response, skin aging, wound healing, gene delivery, and the investigation of skin-related conditions, including scleroderma.

Time of exposure was 24, 72, and 168 hr. We used only negative control that include RPMI 1640 and other supplements in procedure without samples, positive control was not used in this experiment.

Cell suspensions were distributed into 96-well plates, with each well containing 100 *µ*L of the suspension, along with or without the test compounds. Subsequently, the plates were placed in a humidified incubator at 37°C with 5% CO_2_ for the designated duration of exposure.

Following the incubation period, a 10 *µ*L solution of MTT was introduced into each well, resulting in a final concentration of 0.45 mg/mL. Subsequently, the plates were reincubated at 37°C for an additional 1–4 hr. Upon completion of this incubation, the formazan crystals that developed were dissolved in 100 *µ*L of a solubilization solution. The absorbance of the resulting solution was then quantified at 570 nm using a multiplate reader. This measurement allows for the assessment of cell viability and the determination of the cytotoxic effects of the test compounds.

## 3. Results

### 3.1. Antibacterial Activity

**Figure 1:**
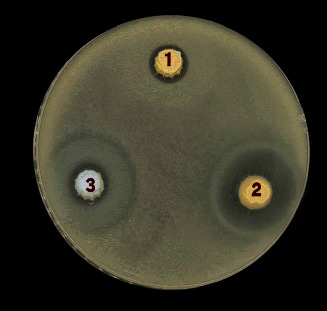
Antibacterial activity of tested materials against *E. Faecalis*. 1—AH Plus; 2—AH Plus + NACP; and 3—NACP.

**Figure 2:**
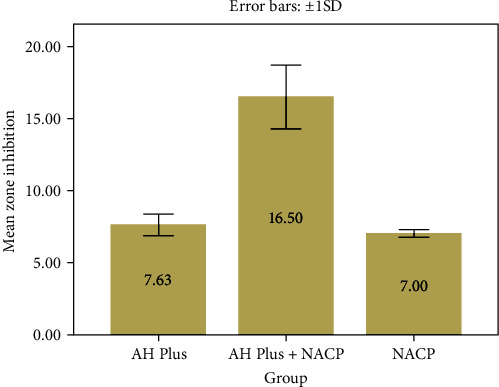
The mean and standard deviation of the inhibition zone diameter (mm) of the different materials used.

In this in vitro study, the antibacterial efficacy of root canal sealers (AH Plus, AH Plus + NACP) and NACP powder against *E. faecalis* strain was evaluated. The agar diffusion test method was employed to assess the antibacterial effect. This test involves observing the formation of an inhibition zone, also known as a halo, surrounding the study material on the agar plate. The extent of this zone directly indicates the extent of the antibacterial effect of the sealer, as illustrated in Table 2 and Figures 1 and 2.

LSD test is one of the post hoc tests used in this study following the ANOVA test to determine which groups were different significantly from each other. AH Plus + NACP was significantly different from both AH Plus and NACP (*P* <0.005). While AH Plus and NACP were not significantly different (*P*=0.394) (Table 3).

### 3.2. Cytotoxicity

**Figure 3:**
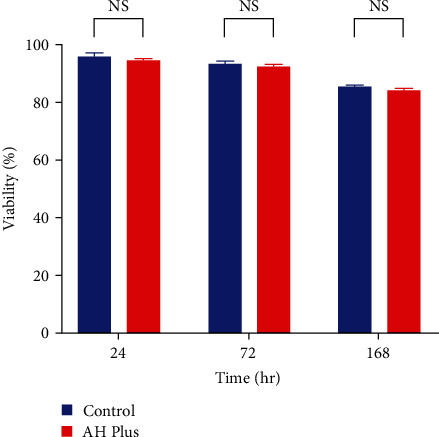
MTT assay bar chart for AH Plus sealer compared with negative control in different time intervals. NS, non-significant.

**Figure 4:**
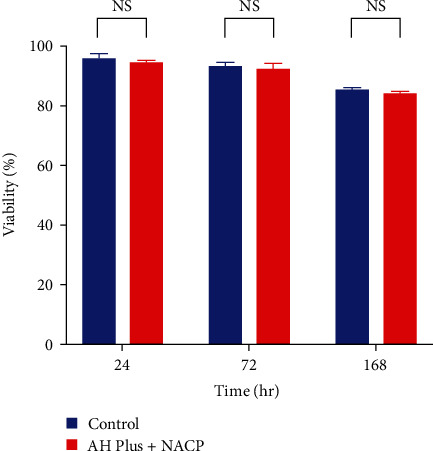
MTT assay bar chart for AH Plus + NACP compared with negative control in different time intervals. NS, non-significant.

**Figure 5:**
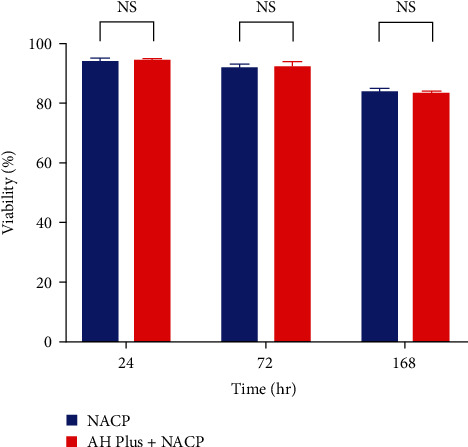
MTT assay bar chart for AH Plus + NACP compared with NACP in different time intervals. NS, non-significant.

In accordance with ISO 10993-5:2009, a decrease in cell viability exceeding 30% is regarded as indicative of cytotoxicity, all tested materials in this study (AH Plus, AH Plus + NACP, and NACP) with different time intervals and concentrations were used, considered not cytotoxic. The results of the MTT assay over all time periods are represented in Figures 3–5. AH Plus and AH Plus + NACP showed no cytotoxic effects after 24, 72 hr, and 1 week of manipulation when compared with negative controls (*P* > 0.05) (Tables 4–6). NACP powder was assessed with different concentrations and time intervals, which also showing no cytotoxic effect, as explained in Tables 7–9.

## 4. Discussion

Before using bioactive additives in human teeth, it is essential to ensure their compatibility with mammalian cells. Previous research has explored the biocompatibility of antibacterial and remineralizing supplements, focusing on evaluations against mouse fibroblasts (L929) [41]. These investigations predominantly centered on their integration into methacrylate resin-based bonding agents and composite materials. Nevertheless, it is plausible to anticipate analogous outcomes when these additives are integrated into epoxy resin-based endodontic sealers.

Researchers used a composite and adhesive system containing DMADDM and NACP to repair occlusal cavities on the first molars of rats in a study [34]. The assessment encompassed an examination of both the pulpal inflammatory response and tertiary dentin formation. Groups that included DMADDM + NACP exhibited superior biocompatibility and reduced tissue disarray when compared to the control groups. Notably, after 30 days, restorations containing NACP showcased a remarkable four- to six-fold increase in tertiary dentin formation compared to the control [42]. In this study, groups containing AH Plus + NACP showed biocompatibility with nosignifficant difference with AH Plus group as well as negative control group.

Previous research has shown that incorporating NACP into dental composites resulted antibacterial properties, reducing the biofilm growth, metabolic activity, and acid production of *S. mutans* biofilms [43]. It is possible that the alkalinity of the NACP can reduce the bacteria growth [44]. The authors also emphasized that these nanocomposites have the potential to provide advantages in terms of both remineralization and antibacterial properties, thereby effectively preventing dental caries [43].

Further studies have reported the antibacterial effects of incorporating NACP into dental materials, specifically on *Actinomyces naeslundii*, *Fusobacterium nucleatum*, and *E. faecalis*. These studies conducted experiments using saliva-coated sealer disks and found that the incorporation of NACP resulted in significant antibacterial activity against these microorganisms [45].

In this study, incorporating NACP with AH Plus sealer significantly increased the antibacterial action of AH Plus sealer without affecting its cytocompatibility and other physical properties. It has been hypothesized that the release of Ca^2+^ ions from NACP particles could induce a toxic effect on bacterial cells, much like the mechanism by which they are believed to degrade eukaryotic cells [46, 47].

In an earlier investigation, scientists discovered that calcium phosphate powder displayed enhanced antibacterial efficacy against *Staphylococcus aureus* bacteria, resulting in inhibition zones that spanned from 0.2 to 0.7 cm [48].

By disrupting crucial molecular mechanisms, nanoparticles (NPs) are able to penetrate the cell wall and membrane of bacteria. When used alongside suitable antibiotics, they may exhibit a synergistic effect, which could potentially aid in mitigating the ongoing issue of bacterial resistance [49].

## 5. Conclusions

This study aimed to create a modified root canal sealer that incorporates NACP, demonstrating contact-killing abilities and facilitating the remineralization and strengthening of dentin. The NACP-infused sealer effectively neutralized acidity, raised pH levels, and released calcium and phosphate ions without compromising flowability. In laboratory tests against *E. faecalis*, the sealer significantly reduced bacterial growth compared to the control. The modified sealer holds potential in enhancing the effectiveness of initial endodontic treatment by precisely addressing lingering bacteria, averting subsequent infections, and releasing calcium and phosphate ions to fortify and safeguard the structure of the tooth root structure.

## Figures and Tables

**Table 1:** Endodontic sealer and nanopowder used in the study.

Material	Manufacturer	Composition
AH Plus	Dentsply, Germany	Epoxide paste. Diepoxide, calcium tungstate, zirconium oxide, aerosil, pigment amine paste. 1-Adamantane amine, N,N′-dibenzyl-5-oxa-nonandiamine-1,9, TCD-diamine, calcium tungstate, zirconium oxide, aerosil, silicone oil

NACP	Sigma–Aldrich, USA	Ca_2_P_2_O_7_._X_H_2_O

**Table 2:** Antibacterial activity (zone of inhibition) of sealers and NACP.

Sample	AH Plus	AH Plus + NACP	NACP
*E. faecalis*	7	12	7
	7	17	7
	8	18	7
	7	19	7.5
	9	18	7
	7	16	7
	8	15	6.5
	8	17	7

Mean	7.63	16.50	7.000

SD	0.74	2.20	0.267

**Table 3:** A one-way ANOVA test and LSD test.

	Group			*P* value ANOVA
	AH Plus	AH Plus + NACP	NACP	
Zone inhibition				
Mean	7.63	16.50	7.00	0.005
SD	0.74	2.20	0.29	
Minimum	7.00	12.00	6.50	
Maximum	9.00	19.00	7.50	

**Table 4:** An independent samples *t*-test was used to determine the significance of differences in cell viability mean values between the AH Plus sealer group and the negative control group at different time points.

Time (hr)	Mean viability (%) ± SD		*P* value (*t*-test)	Significance
	Negative control	AH Plus		
24	95.563 ± 1.50	94.213 ± 1.13	0.3599	NS
72	93.17 ± 1.0	92.168 ± 1.037	0.5959	NS
168	85.339 ± 0.58	84.0663 ± 0.70	0.4071	NS

NS, non-significant.

**Table 5:** To assess the significance of variations in the mean cell viability values, an independent samples *t*-test was performed between the AH Plus + NACP sealer group and the negative control group at different time intervals.

Time (hr)	Mean viability (%) ± SD		*P* value (*t*-test)	Significance
	Negative control	AH Plus + NACP		
24	95.563 ± 1.50	94.251 ± 0.582	0.4386	NS
72	93.17 ± 1.0	92.129 ± 1.75	0.6182	NS
168	85.339 ± 0.58	83.564 ± 0.64	0.2080	NS

NS, non-significant.

**Table 6:** An independent samples *t*-test was conducted to assess the statistical significance of variations in the mean cell viability values between the AH Plus sealer group and the AH Plus + NACP sealer group at different time intervals.

Time (hr)	Mean viability (%) ± SD		*P* value (*t*-test)	Significance
	AH Plus	AH Plus + NACP		
24	94.213 ± 1.13	94.251 ± 0.582	>0.9999	NS
72	92.16 ± 1.03	92.129 ± 1.75	>0.9999	NS
168	84.06 ± 0.70	83.564 ± 0.64	0.9214	NS

NS, non-significant.

**Table 7:** The cytotoxic effect of NACP on HdFn cell line at 24 hr.

Concentration (*µ*g mL^−1^)	Mean viability (%) ± SD
	HdFn
200	82.60 ± 2.3
100	86.07 ± 3.07
50	90.08 ± 1.04
25	93.86 ± 1.10
12.5	94.63 ± 0.48
6.25	95.10 ± 0.29
3.125	96.33 ± 0.41

**Table 8:** The cytotoxic effect of NACP on HdFn cell line at 72 hr.

Concentration (*µ*g mL^−1^)	Mean viability (%) ± SD
	HdFn
200	73.148 ± 1.13
100	80.94 ± 4.52
50	92.284 ± 0.99
25	96.33 ± 0.40
12.5	96.29 ± 0.8
6.25	97.10 ± 1.0
3.125	97.39 ± 0.64

**Table 9:** The cytotoxic effect of NACP on HdFn cell line at 168 hr.

Concentration (*µ*g mL^−1^)	Mean viability (%) ± SD
	HdFn
200	71.95 ± 0.81
100	76.69 ± 2.60
50	84.33 ± 2.66
25	86.07 ± 1.91
12.5	95.216 ± 0.82
6.25	95.949 ± 1.02
3.125	95.978 ± 1.14

## Data Availability

The data that support the findings of this study are available in Phi Nano-Science Center (PNSC), Baghdad, Iraq, info@phi-nano.com, tel. +(964)7832847564, and the Centre of Natural Product Research and Drug Discovery, University of Malaya, Malaysia.

## Additional Points

*Clinical implication*. Incorporation NACP into epoxy resin AH Plus sealer holds promise for delivering dual advantages, offering both remineralization and antibacterial properties to effectively hinder bacterial growth within the root canal system. *Limitation of study. Cytotoxicity*. We used cell line not animal test, the last one could be more reliable than the first one. *Antibacterial study*. This is an in vitro study used agar media not human teeth, also, in this experiment, we used diffusion test that show the effect of the modified sealer on planktonic bacteria rather than biofilm; the most popular pathogen pattern found in root canal dentin walls.
